# Supplementary material for: In Vitro Activity of Selected Phenolic Compounds against Planktonic and Biofilm Cells of Food-Contaminating Yeasts
Source: Foods. 2021 Jul 17;10(7):1652. doi: 10.3390/foods10071652 (PMC8307438; doi:10.3390/foods10071652)
Supplement: Supplementary file 1 [file foods-10-01652-s001.zip › Supplementary material_Kimani.pdf]

Supplementary material

In vitro activity of selected phenolic compounds against planktonic and biofilm cells of food-contaminating yeasts

Bernard Gitura Kimani [1], Erika Beáta Kerekes [1], Csilla Szebenyi [1,2], Judit Krisch [3], Csaba Vágvölgyi [1], Tamás Papp [1,2] and Miklós Takó [1,*]

[1] Department of Microbiology, Faculty of Science and Informatics, University of Szeged, Közép fasor 52, H-6726 Szeged, Hungary

[2] MTA-SZTE "Lendület" Fungal Pathogenicity Mechanisms Research Group, University of Szeged, Közép fasor 52, H-6726 Szeged, Hungary

[3] Institute of Food Engineering, Faculty of Engineering, University of Szeged, Mars tér 7, H-6724 Szeged, Hungary

*Correspondence: tako78@bio.u-szeged.hu; Tel.: +36-62-544-516

List of figures

Figure S1. Effect of phenolic compounds on the growth of *D. hansenii* SZMC 8045Mo (A), *P. anomala* SZMC 8061Mo (B), *S. pombe* SZMC 1280 (C) and *S. cerevisiae* SZMC 1279 (D) at a concentration of 1 mg/mL.

Figure S2. Effect of phenolic compounds on the growth of *D. hansenii* SZMC 8045Mo (A), *P. anomala* SZMC 8061Mo (B), *S. pombe* SZMC 1280 (C) and *S. cerevisiae* SZMC 1279 (D) at a concentration of 500 μg/mL.

Figure S3. Effect of phenolic compounds on the growth of *D. hansenii* SZMC 8045Mo (A), *P. anomala* SZMC 8061Mo (B), *S. pombe* SZMC 1280 (C) and *S. cerevisiae* SZMC 1279 (D) at a concentration of 250 μg/mL.

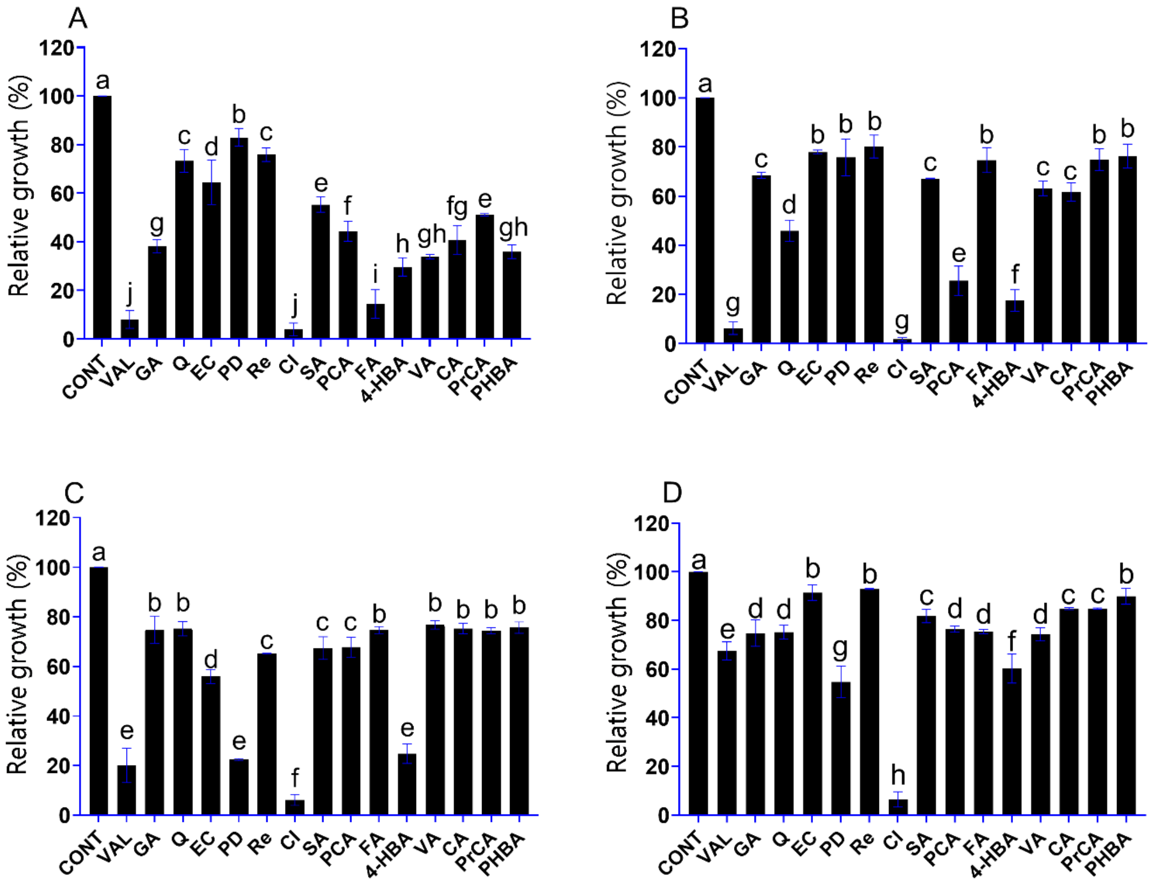

Figure S1. Effect of phenolic compounds on the growth of *D. hansenii* SZMC 8045Mo (A), *P. anomala* SZMC 8061Mo (B), *S. pombe* SZMC 1280 (C) and *S. cerevisiae* SZMC 1279 (D) at a concentration of 1 mg/mL. Phenolic compounds: vanillin (VAL), gallic acid (GA), quercetin (Q), (−)-epicatechin (EC), polydatin (PD), resveratrol (Re), cinnamic acid (CI), syringic acid (SA), *p*-coumaric acid (PCA), ferulic acid (FA), 4-hydroxybenzaldehyde (4-HBA), vanillic acid (VA), caffeic acid (CA), protocatechuic acid (PrCA), 4-hydroxybenzoic acid (PHBA). The control (CONT) represents the growth of yeasts in the absence of phenolic compounds. Presented results are averages of three biological and three technical replicates; error bars represent standard deviations. Different letters indicate statistical differences according to one-way ANOVA followed by Tukey's multiple comparison test ($p < 0.05$).

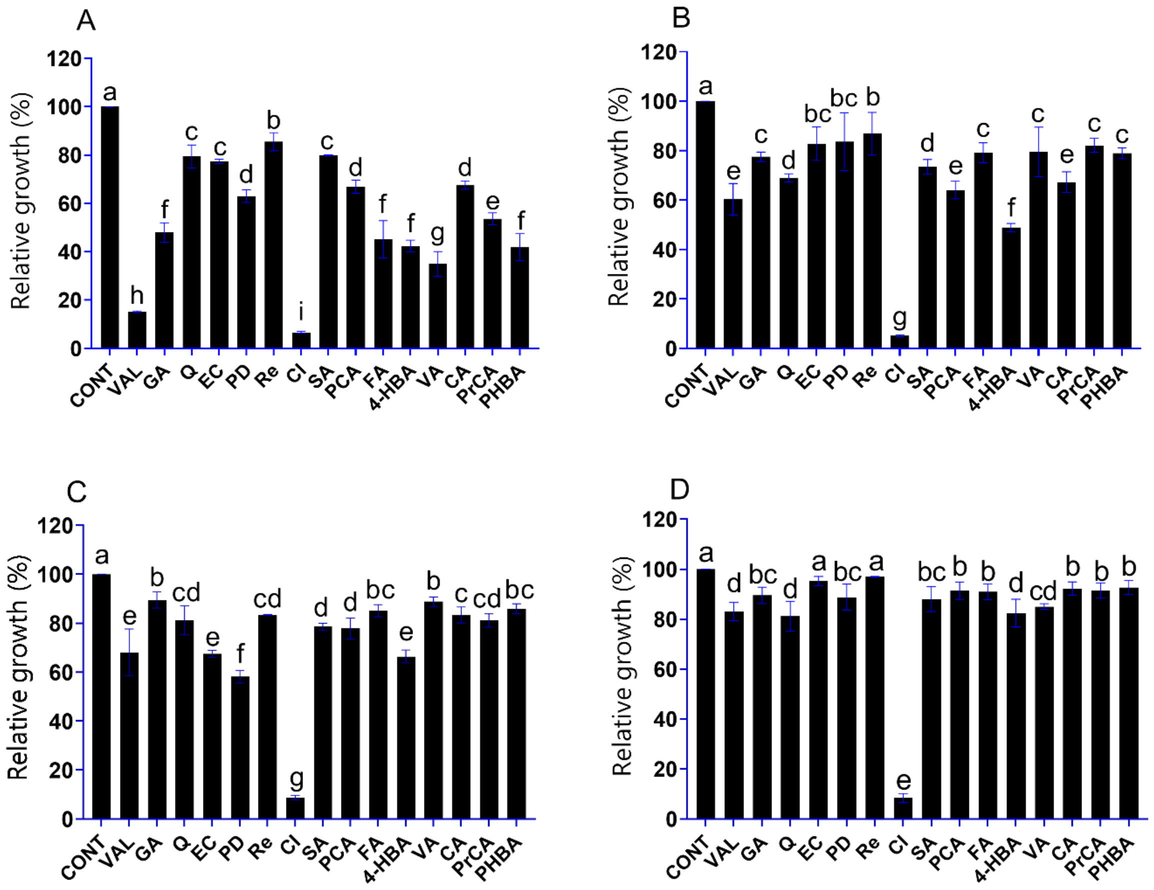

Figure S2. Effect of phenolic compounds on the growth of *D. hansenii* SZMC 8045Mo (A), *P. anomala* SZMC 8061Mo (B), *S. pombe* SZMC 1280 (C) and *S. cerevisiae* SZMC 1279 (D) at a concentration of 500 µg/mL. Phenolic compounds: vanillin (VAL), gallic acid (GA), quercetin (Q), (−)-epicatechin (EC), polydatin (PD), resveratrol (Re), cinnamic acid (CI), syringic acid (SA), *p*-coumaric acid (PCA), ferulic acid (FA), 4-hydroxybenzaldehyde (4-HBA), vanillic acid (VA), caffeic acid (CA), protocatechuic acid (PrCA), 4-hydroxybenzoic acid (PHBA). The control (CONT) represents the growth of yeasts in the absence of phenolic compounds. Presented results are averages of three biological and three technical replicates; error bars represent standard deviations. Different letters indicate statistical differences according to one-way ANOVA followed by Tukey's multiple comparison test ($p < 0.05$).

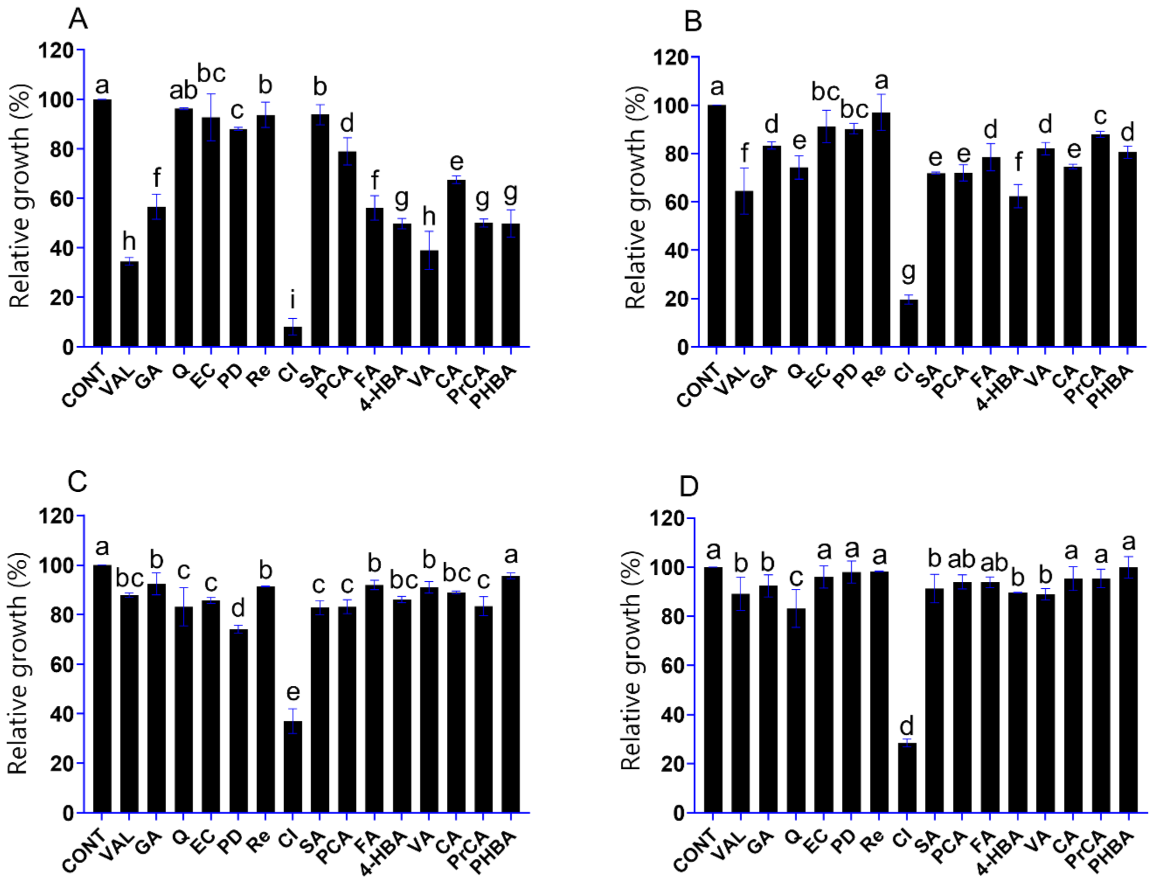

Figure S3. Effect of phenolic compounds on the growth of *D. hansenii* SZMC 8045Mo (A), *P. anomala* SZMC 8061Mo (B), *S. pombe* SZMC 1280 (C) and *S. cerevisiae* SZMC 1279 (D) at a concentration of 250 µg/mL. Phenolic compounds: vanillin (VAL), gallic acid (GA), quercetin (Q), (−)-epicatechin (EC), polydatin (PD), resveratrol (Re), cinnamic acid (CI), syringic acid (SA), *p*-coumaric acid (PCA), ferulic acid (FA), 4-hydroxybenzaldehyde (4-HBA), vanillic acid (VA), caffeic acid (CA), protocatechuic acid (PrCA), 4-hydroxybenzoic acid (PHBA). The control (CONT) represents the growth of yeasts in the absence of phenolic compounds. Presented results are averages of three biological and three technical replicates; error bars represent standard deviations. Different letters indicate statistical differences according to one-way ANOVA followed by Tukey's multiple comparison test ($p < 0.05$).
